# PARTICIPANT EVALUATION OF A SOCIAL ENGAGEMENT PROGRAM FOR FAMILY CARE PARTNERS OF PERSONS WITH DEMENTIA

**DOI:** 10.1093/geroni/igae098.3379

**Published:** 2024-12-31

**Authors:** Eliza Baby, Elizabeth Lydon, Sarah Jones, Xiayu Chen, Vincent F Mathias, Wendy Rogers, Minakshi Raj, Raksha Mudar

**Affiliations:** University of Illinois Urbana-Champaign, Urbana, Illinois, United States; University of Illinois Urbana-Champaign, Champaign, Illinois, United States; University of Illinois Urbana-Champaign, Champaign, Illinois, United States; University of Illinois Urbana-Champaign, Champaign, Illinois, United States; University of Illinois Urbana-Champaign, Champaign, Illinois, United States; University of Illinois Urbana-Champaign, Champaign, Illinois, United States; University of Illinois Urbana-Champaign, Champaign, Illinois, United States; University of Illinois Urbana-Champaign, Champaign, Illinois, United States

## Abstract

Caregivers of persons with dementia (PwD) often experience increased social isolation, loneliness, and negative health outcomes. While support groups provide critical education and connection for care partners, there is less opportunity for engagement outside of the caregiving role. We developed and assessed the benefits of a technology-based social engagement program, CareEngage, designed to be convenient and accessible to older adult caregivers (60+ years) of a family member with dementia. Sixty-four older caregivers completed the intervention. Participants attended eight social engagement sessions over 4-6 weeks. During each session, participants met remotely via Zoom for casual, unmoderated conversations on various topics of interest. The topics were intentionally unrelated to caregiving and covered five categories: Arts and Culture; Life Experiences; Nature, Health, and Wellness; Recreation and Sports; and Science and Technology. Immediately following each session, participants completed a survey to assess self-reported enjoyment, engagement, and connection on a five-point scale in addition to providing an open-ended comment. Participants also completed a post-program survey to capture overall program satisfaction. A mixed methods approach was employed to assess post-session and post-program surveys. High scores were reported on enjoyment (mean = 4.41 ± 0.89), engagement (mean = 4.52 ± 0.78), and connection (mean = 4.44 ± 0.84). On the post-program survey, the majority reported they enjoyed the program (75%), and they would recommend it to others (72%). Findings suggest virtual social engagement opportunities may be beneficial for enhancing the social well-being of older care partners of PwD.

